# Simulated Microgravity Reduces Focal Adhesions and Alters Cytoskeleton and Nuclear Positioning Leading to Enhanced Apoptosis via Suppressing FAK/RhoA-Mediated mTORC1/NF-κB and ERK1/2 Pathways

**DOI:** 10.3390/ijms19071994

**Published:** 2018-07-08

**Authors:** Tuo Zhao, Rong Li, Xin Tan, Jun Zhang, Cuihong Fan, Qin Zhao, Yulin Deng, Aizhang Xu, Kiven Erique Lukong, Harald Genth, Jim Xiang

**Affiliations:** 1 School of Life Sciences, Beijing Institute of Technology, Beijing 10081, China; zhaotuobeijing@hotmail.com (T.Z.); tanxing@bit.edu.cn (X.T.); zhangjun@bit.edu.cn (J.Z.); chfan@bit.edu.cn (C.F.); zhaoqin@hotmail.com (Q.Z.); deng@bit.edu.cn (Y.D.); 2 Cancer Research, Saskatchewan Cancer Agency, Saskatoon, SK S7N 4H4, Canada; rol840@mail.usask.ca (R.L.); aix705@mail.usask.ca (A.X.); 3 Department of Oncology, University of Saskatchewan, Saskatoon, SK S7N 5E5, Canada; 4 Department of Biochemistry, University of Saskatchewan, Saskatoon, SK S7N 5E5, Canada; Kiven.lukong@usask.ca; 5 Institute of Toxicology, Hannover Medical School, D-30625 Hannover, Germany; Genth.Harald@mh-hannover.de

**Keywords:** SMG, apoptosis, focal adhesion complex, cytoskeleton, nuclear positioning, FAK, RohA, mTORC1, NK-κB, ERK1/2

## Abstract

Simulated-microgravity (SMG) promotes cell-apoptosis. We demonstrated that SMG inhibited cell proliferation/metastasis via FAK/RhoA-regulated mTORC1 pathway. Since mTORC1, NF-κB, and ERK1/2 signaling are important in cell apoptosis, we examined whether SMG-enhanced apoptosis is regulated via these signals controlled by FAK/RhoA in BL6-10 melanoma cells under clinostat-modelled SMG-condition. We show that SMG promotes cell-apoptosis, alters cytoskeleton, reduces focal adhesions (FAs), and suppresses FAK/RhoA signaling. SMG down-regulates expression of mTORC1-related Raptor, pS6K, pEIF4E, pNF-κB, and pNF-κB-regulated Bcl2, and induces relocalization of pNF-κB from the nucleus to the cytoplasm. In addition, SMG also inhibits expression of nuclear envelope proteins (NEPs) lamin-A, emerin, sun1, and nesprin-3, which control nuclear positioning, and suppresses nuclear positioning-regulated pERK1/2 signaling. Moreover, rapamycin, the mTORC1 inhibitor, also enhances apoptosis in cells under 1 g condition via suppressing the mTORC1/NF-κB pathway. Furthermore, the FAK/RhoA activator, toxin cytotoxic necrotizing factor-1 (CNF1), reduces cell apoptosis, restores the cytoskeleton, FAs, NEPs, and nuclear positioning, and converts all of the above SMG-induced changes in molecular signaling in cells under SMG. Therefore, our data demonstrate that SMG reduces FAs and alters the cytoskeleton and nuclear positioning, leading to enhanced cell apoptosis via suppressing the FAK/RhoA-regulated mTORC1/NF-κB and ERK1/2 pathways. The FAK/RhoA regulatory network may, thus, become a new target for the development of novel therapeutics for humans under spaceflight conditions with stressed physiological challenges, and for other human diseases.

## 1. Introduction

The spaceflight environment, containing many physical and psychological stress factors, severely affects human physiology and health [1]. One of the physiological stress factors is microgravity, which has been demonstrated to promote cell apoptosis [2,3,4]. Apoptosis is a cell-programmed death with characteristics of cell shrinkage, chromatin condensation, nuclear collapse and fragmentations, and cytoplasmic blebbing leading to the formation of apoptotic bodies [5]. It has been shown that multiple pathways regulate the formation of cell apoptosis, including the mammalian target of rapamycin complex (mTORC), nuclear factor-kappa B (NF-κB), extracellular signal-regulated kinase-1/2 (ERK1/2), and P53/PUMA [6].

The cytoskeleton, a cellular structural scaffold, functions in maintaining the cell shape, providing an intracellular transport system, regulating cell migration, and controlling cell survival [7]. The eukaryotic cytoskeleton is composed of intermediate filaments, actin filaments, and microtubules. Cell surface integrins and intracellular cytoskeleton interact with the extracellular matrix at cellular membrane sites, termed focal adhesions (FAs) [8]. The integrin-binding molecules, such as talin, vinculin, and paxillin, recruit focal adhesion kinase (FAK) to the site of focal adhesions to form focal adhesion complexes (FACs), which also comprise another group of the ras homolog gene (Rho) family GTPases [9]. The Rho family members, including Rho family member-A (RhoA), cell division-control protein-42 (Cdc42), and ras-related C3 botulinum-toxin substrate-1 (Rac1), control the function of actin-binding proteins function to form higher-order structures, such as stress fibers (actin/myosin bundles), lamellipodia (membrane ruffles at the leading edge), and filopodia (membrane protrusion) [10]. In addition, the Rho family members also control some molecular signaling such as the mTORC1 [11,12,13]. Signaling FAK and RhoA have also been found to protect cells from apoptosis and mediate cell survival [14,15]. 

Nuclei, though often localized in the center of cells, are sometimes positioned asymmetrically depending upon the status of cell differentiation and migration, as well as the stage of cell cycling [16]. The molecular toolbox elements controlling nuclear positioning include cytoskeleton and nucleoskeleton, as well as nuclear envelope protein complexes (NEPCs) [16]. Nuclear positioning has been shown to affect cytoplasmic signaling and influence the nucleus to access signaling pathways, such as RhoA and ERK1/2 pathways [17,18].

Simulated microgravity (SMG), a ground-based method developed to mimic the gravitational conditions that exist in space, was reported to alter cytoskeleton structures [13,19,20,21] and induce cell apoptosis via up- and down-regulated expression of pro-apoptosis and anti-apoptosis molecules [3,4]. We previously applied a random positional machine modeling SMG to investigate SMG’s effect on cell apoptosis and its underlying molecular mechanism [2]. We demonstrated that SMG altered cytoskeleton and promoted cell apoptosis through suppressing NF-κB pathway leading to up- and down-regulated pro-apoptosis (caspases 3, 7, 8) and anti-apoptosis (Bcl2 and Bnip3) molecules, respectively [2]. However, the up-stream molecules regulating the NF-κB-regulated anti-apoptosis effect in cells under SMG condition is still unclear. We have recently demonstrated that SMG reduced Fas, leading to reduced cell proliferation, invasiveness, and metastasis via inhibition of the FAK/RhoA-regulated mTORC1 pathway [22].

In this study, we investigated SMG’s effects on the cytoskeleton structure and formation of FAs. We also examined a potential molecular mechanism regulating the SMG-induced cell apoptosis through studying the cytoskeleton structure, Fas, and expression of signaling molecules in FAs, such as FAK and Rho family proteins (RhoA, Rac1, and Cdc42) and mTORC1 pathway molecules, such as S6 kinase (S6K) and eukaryotic initiation factor-4E (EIF4E) in cells under SMG. In addition, we further assessed SMG’s effect on nuclear positioning through examining the expression of NEPCs in cells under SMG by fluorescence microscopy and Western blotting analyses. We found that SMG reduced FAs and altered the cytoskeleton and nuclear positioning, leading to enhanced cell apoptosis via suppressing FAK/RhoA-controlled mTORC1/NF-κB and ERK1/2 pathways.

## 2. Results

### 2.1. Simulated Microgravity Promotes Cell Apoptosis

**Figure 1:**
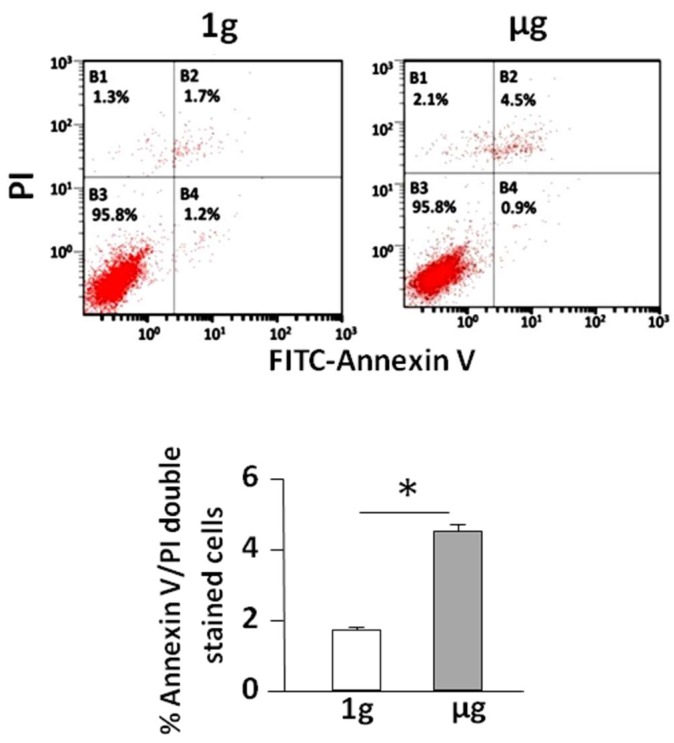
Simulated microgravity induces BL6-10 cell apoptosis. BL6-10 tumor cells cultured underground condition (1 g) and SMG (µg) for 24 h were stained with Annexin V-FITC and propidium iodide (PI), and then analyzed by flow cytometry. Data represent the mean ± SD of three independent experiments. * *p*< 0.05 versus different groups.

Previous reports from our own or other laboratories demonstrated that SMG promotes cell apoptosis [2,3,4]. In this study, we analyzed B16 melanoma BL6-10 cell apoptosis in cells under SMG using an apoptosis kit, and observed apoptotic cells in late stage that were positive for both Annexin V and propidium iodide (PI) (Figure 1). Under SMG (µg) conditions 4.5% the cells were apoptotic, which is significantly more than the 1.7% observed under 1 g conditions (Figure 1), thus indicating that SMG promotes BL6-10 cell apoptosis.

### 2.2. Simulated Microgravity Alters the Cytoskeleton Structure and Inhibits Expression of F-Actin and F-Actin-Binding Protein

**Figure 2:**
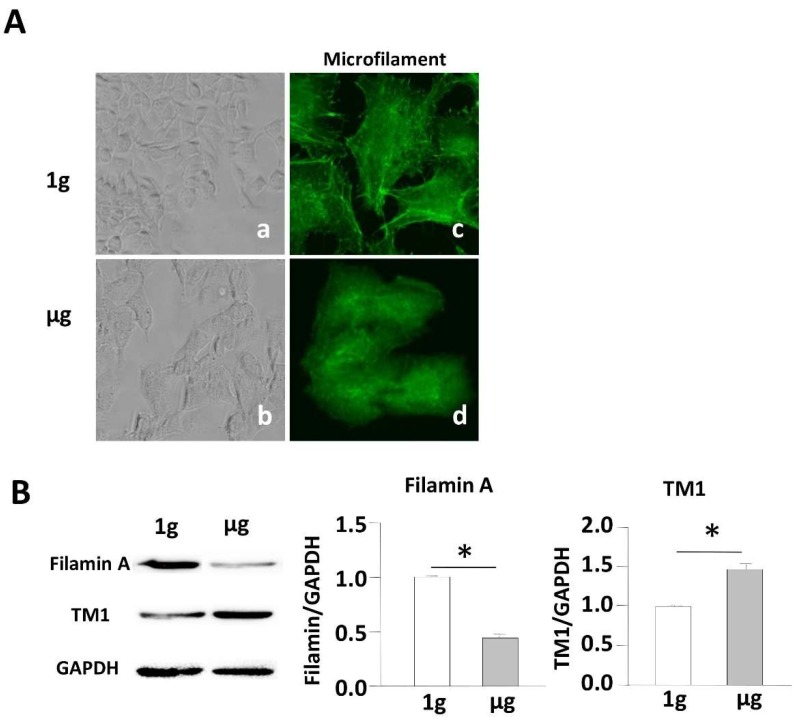
Simulated microgravity alters the cytoskeleton and inhibits the expression of F-actin and actin-binding protein. (**A**) BL6-10 cells were cultured in chamber slides for one day at 1 g or µg. The cells were also stained with FITC-phalloidin (green) and analyzed by light microscopy and fluorescence microscopy. Panels (**a**,**b**) using 10× magnification; panels (**c**,**d**) using 40× magnification; (**B**) Lysates prepared from BL6-10 cells cultured for three days at 1 g or µg were subjected to SDS-PAGE analysis. Proteins were transferred onto PVDF membranes and blotted with the indicated antibodies. Western blot band signals were quantified by chemiluminescence. Densitometric values were normalized to matching GAPDH controls. Data represent the mean ± SD. * *p* < 0.05 versus different groups. One representative experiment of three is shown.

Morphological changes of the apoptotic cell are mediated by several proteins of the cytoskeleton. Previous reports from our own or other laboratories demonstrated that SMG alters cytoskeleton structures [2,13,19,20,22]. BL6-10 cells growing on the surface of culture chamber slides under 1 g conditions displayed flat and irregular morphology, while they became thick and rounded in shape and aggregated into clusters under SMG (Figure 2A(a,b)), indicating that their cytoskeleton structures may be altered under SMG. To assess the cytoskeleton alteration triggered by SMG, we stained cells with FITC-labeled phalloidin for the measurement of microfilaments. Cells under 1 g conditions showed an even distribution of staining, abundant lamellipodia, stress fibers, and filopodia, while cells under SMG showed a dramatic decrease in lamellipodia and stress fibers (Figure 2A(c,d)), which is consistent with our previous report [2]. These data further validate that SMG alters the cytoskeleton structure. F-actin filamin-A and F-actin-binding proteins (ABPs) such as TM1, are key components of actin filaments, and have been found to regulate cell apoptosis [23]. We, therefore, assessed the effect of SMG on the expression of ABPs by Western blotting analysis using anti-filamin-A and anti-TM1 antibodies. We observed that expression levels of filamin-A and TM1 were down-regulated and up-regulated, respectively, in cells under SMG than that in cells under 1 g conditions (Figure 2B), suggesting that these molecules may play a role in SMG-mediated cytoskeletal changes.

### 2.3. Simulated Microgravity Reduces Focal Adhesions and Inhibits FAK and RhoA Signaling

**Figure 3:**
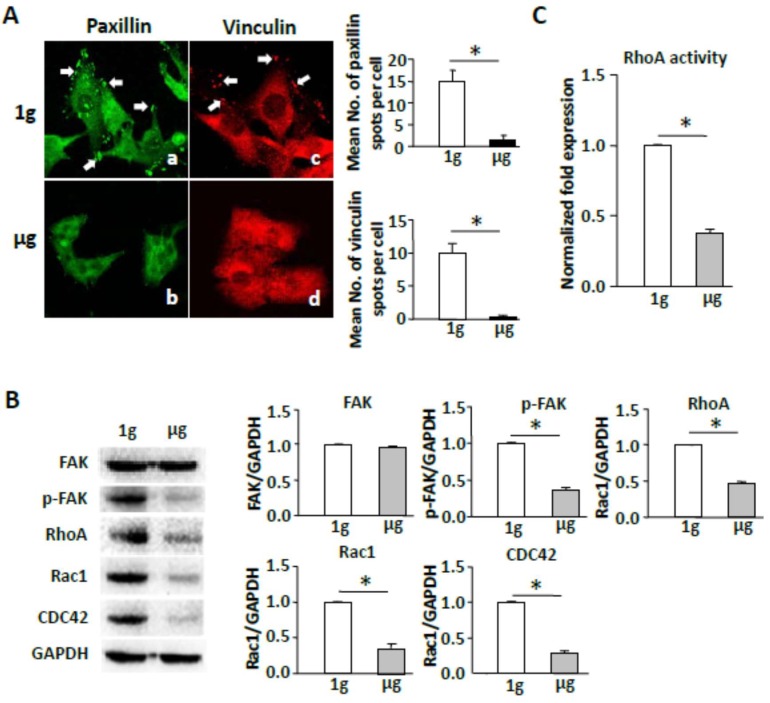
Simulated microgravity reduces focal adhesions and inhibits FAK and RhoA signaling. (**A**) BL6-10 cells were cultured in medium of chamber slides for one day under ground conditions (1 g) or SMG (µg). The cells were stained with anti-paxillin (green) and anti-vinculin (red) antibodies followed by observation under a fluorescence microscope using 40× objectives (formation of cellular focal adhesions (a,c), arrows); (**B**) Western blotting analysis. Lysates were harvested from BL6-10 cells cultured for three days under 1 g or µg and subjected to SDS-PAGE analysis. Proteins were transferred onto PVDF membranes. Blots were stained with various antibodies and analyzed by chemiluminescence. Bands were qualified using Imaging Lab software (Bio-Rad). Densitometric values were normalized to the GAPDH control; (**C**) RhoA activity analysis. BL6-10 cells (three days) under 1 g and µg were subjected to RhoA activity assay by using a G-LISA RhoA Activation Assay Biochem kit. Data represent the mean ± SD of three independent experiments. * *p* < 0.05 versus different groups. One representative experiment of two is shown.

Since integrin-binding proteins, which recruit FAK to focal adhesions, are a part of cellular FAs [9], we investigated the effect of SMG on the integrity of integrin-binding proteins. We stained cells grown on slides coated with integrin ligand fibronectin under SMG with anti-integrin-binding protein paxillin antibody, followed by examination of the cells by fluorescence microscopy to assess the formation of cell FAs. We found that the total number of FAs was substantially reduced in cells under SMG in comparison with control cells under 1 g conditions (Figure 3A). These data indicate that SMG dramatically reduces formation of cellular focal adhesions. FAK is a cytoplasmic tyrosine kinase involved in integrin-mediated signaling and colocalizes with integrins in focal contacts. FAK activation (indicated by phosphorylation at Tyr 397) has been shown to regulate integrins binding to their extracellular ligands. We, therefore, assessed the effect of SMG on FAK activity. We performed Western blotting analysis using cell lysates and anti-FAK and anti-pFAK (Y397) antibodies. We found that the active form of FAK, FAK pY397, was significantly reduced in cells under SMG (1µg), though its FAK expression maintained at the same level as cells under 1 g conditions (Figure 3B). Rho family molecules, including RhoA, Rac1, and Cdc42, are key regulators of cellular morphogenesis and cell polarity mainly through their effects on the cytoskeleton. To determine any functional link between SMG and Rho family molecules, we assessed the effect of SMG on the expression of Rho family proteins by Western blotting analysis using antibodies against RhoA, Rac1, and Cdc42. We showed that SMG induces the down-regulation of RhoA, Rac1, and Cdc42 (Figure 3B). We further assessed the effect of SMG on RhoA activity, using a G-LISA RhoA Activation Assay Biochem kit. We observed that RhoA activity was significantly reduced in SMG-treated cells compared to that in cells under 1 g conditions (Figure 3C). Together, our data indicate that SMG promotes the inhibition of both FAK and RhoA signaling events.

### 2.4. Simulated Microgravity Suppresses the mTORC/NF-κB Pathway

**Figure 4:**
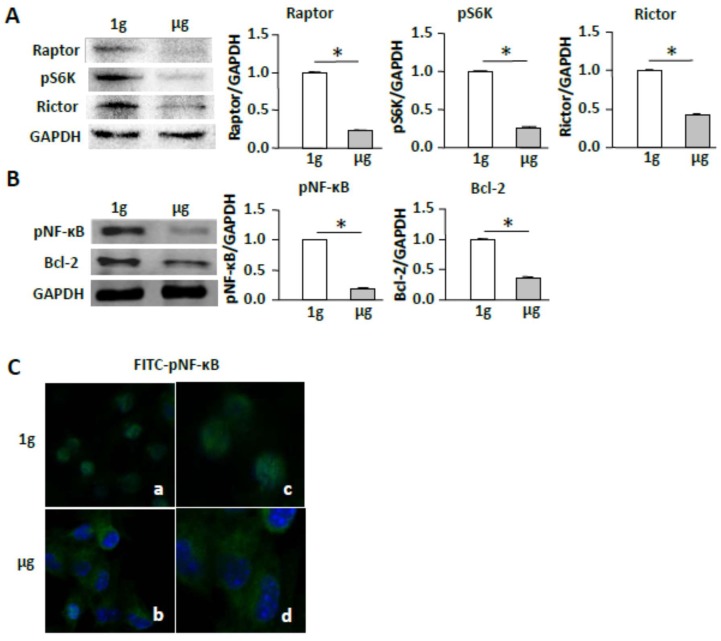
Simulated microgravity suppresses the mTORC/NF-κB pathway. (**A**,**B**) Lysates prepared from BL6-10 cells cultured for two days at 1 g or µg were subjected to SDS-PAGE analysis. Proteins were transferred onto PVDF membranes and blotted with indicated antibodies. Western blot band signals were quantified by chemiluminescence. Densitometric values were normalized to matching GAPDH controls. Data represent the me an ± SD of three independent experiments; (**C**) BL6-10 cells cultured for three days at 1 g or µg in a Lab-Tek1 II Chamber Slide^TM^ System were fixed with paraformaldehyde, and subsequently incubated with anti-p-NF-κB (S337) (green) antibody and then incubated with FITC-labeled goat-anti-rabbit secondary antibody. Slides were covered using Prolong Gold Antifade Reagent with DAPI (blue) and observed by confocal microscopy. Panels (**a**,**b**) using 20× magnification; panels (**c**,**d**) using 50× magnification. * *p* < 0.05 versus different groups. One representative experiment of three is shown.

Since RhoA regulates the conserved signaling the mTORC1 pathway [11,24], we then investigated whether SMG affects the mTORC pathway by assessing the expression of mTORC1-comprising molecule Raptor and mTORC1-regulated molecules, such as S6K and EIF4E [25], as well as mTORC2-comprising molecule Rictor. Interestingly, we found that SMG suppressed the expression of Raptor, pS6K (S235), and Rictor (Figure 4A). Since the mTORC1 regulates NF-κB-mediated cell apoptosis via up-regulation of anti-apoptosis Bcl2 molecule [26], we also assessed the expression of these molecules in cells under SMG. We found that SMG down-regulated expression of pNF-κB (S337) and Bcl2 (Figure 4B), and switched cellular localization of FITC-pNF-κB (S337) (green) from the nucleus (DAPI, blue) to the cytoplasm in cells under SMG by confocal microscopic analysis (Figure 4C).

### 2.5. Rapamycin Inhibits the mTORC1/NF-κB Pathway Leading to Enhanced Apoptosis in Cells under 1 g Conditions

**Figure 5:**
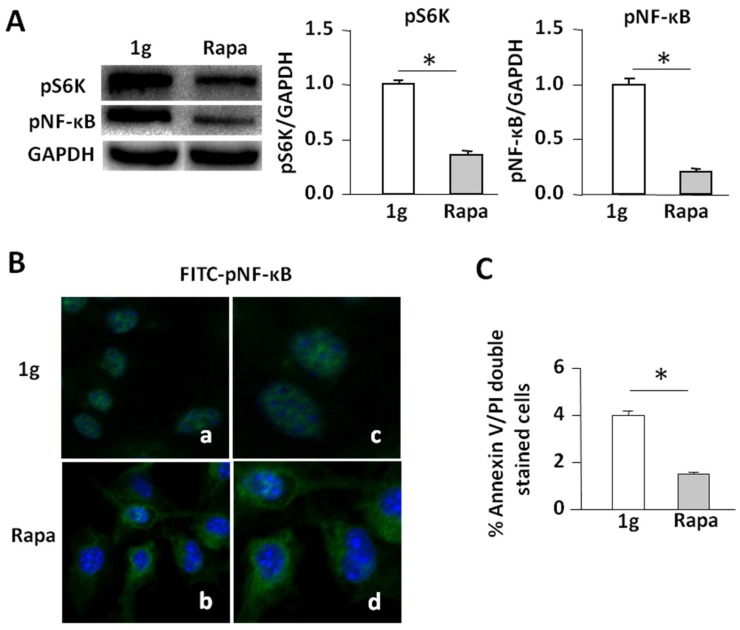
Rapamycin inhibits the mTORC1/NF-κB pathway resulting in the enhancement of apoptosis in cells under the 1 g condition. (**A**) Western blotting analysis. Lysates were harvested from BL6-10 cells cultured for three days under 1 g or 1 g +rapamycin and subjected to SDS-PAGE analysis. Proteins were transferred onto PVDF membranes. Blots were stained with various antibodies and analyzed by chemiluminescence. Bands were qualified using Imaging Lab software (Bio-Rad). Densitometric values were normalized to the GAPDH control. Data represent the mean ± SD of three independent experiments; (**B**) Confocal microscopy analyses. BL6-10 cells cultured for two days under 1 g or 1 g +rapamycin were observed by confocal microscopy. Panels (**a**,**b**) using 20× magnification; panels (**c**,**d**) using 50× magnification. One representative experiment of three is shown; (**C**) BL6-10 tumor cells cultured under ground conditions (1 g) and SMG (µg) for 24 h were stained with Annexin V-FITC and propidium iodide (PI), and then analyzed by flow cytometry. Data represent the mean ± SD of three independent experiments. * *p* < 0.05 versus different groups.

Since rapamycin inhibits the mTORC1/NF-κB pathway [26,27], we performed experiments to assess whether rapamycin mimics the effect of SMG on the promotion of cell apoptosis via suppression of the mTORC1/NF-κB pathway. We found that administration of rapamycin inhibited pS6K (S235) and pNF-κB (S337) (Figure 5A), switched the cellular localization of FITC-pNF-κB (S337) (green) from the nucleus (DAPI, blue) to the cytoplasm (Figure 5B), and increased apoptosis in cells under 1 g conditions (Figure 5C), thus confirming that SMG-induced inhibition of the mTORC1/NF-κB pathway promotes cell apoptosis.

### 2.6. Simulated Microgravity Reduces Nuclear Positioning and Down-Regulated ERK1/2 Pathway

**Figure 6:**
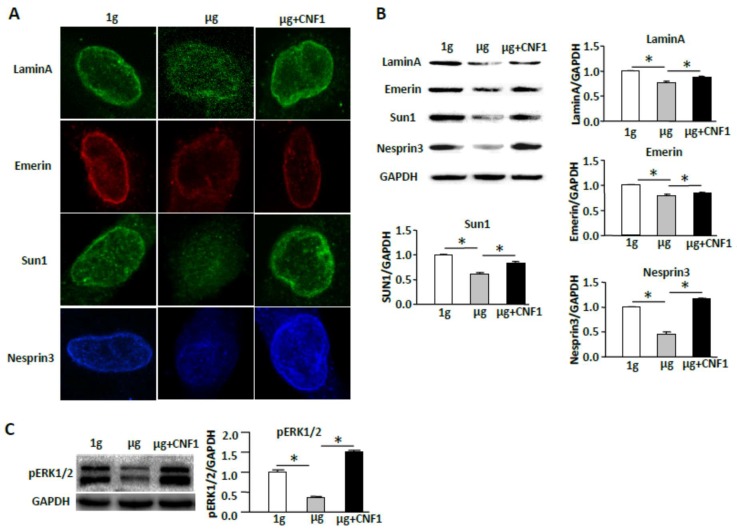
Simulated microgravity decreases nuclear positioning and down-regulates the ERK1/2 pathway. (**A**) BL6-10 cells cultured for two days at 1 g, µg, or µg + CNF1 in a Lab-Tek1 II Chamber Slide^TM^ System were fixed with paraformaldehyde, and subsequently incubated with indicated antibodies and then incubated with FITC-labeled secondary antibodies, and observed by confocal microscopy (300× magnification). One representative experiment of three is shown; (**B**,**C**) Lysates prepared from BL6-10 cells cultured for two days at 1 g, µg, or µg + CNF1 were subjected to SDS-PAGE analysis. Proteins were transferred onto PVDF membranes and blotted with indicated antibodies. Western blot band signals were quantified by chemiluminescence. Densitometric values were normalized to matching GAPDH controls. Data represent the mean ± SD of three independent experiments. * *p* < 0.05 versus different groups.

As mentioned earlier, nuclear positioning has been shown to be regulated by various factors, including the cytoskeleton and nucleoskeleton and nuclear envelope protein complexes [16]. Therefore, to assess whether SMG affects nuclear positioning, we examined nuclear envelop proteins (NEPs) by fluorescence microscopy and Western blotting analyses. We demonstrated that SMG significantly reduced the expression of NEPs (lamin A, emerin, sun1, and nesprin3) on the nuclear membrane by fluorescence microscopy (Figure 6A) and in the cell lysates by Western blotting (Figure 6B), compared to those in cells under the 1 g condition. Our data indicate that SMG alters nuclear positioning via down-regulation expression of NEPs. Since nuclear positioning has been shown to affect ERK1/2 signaling [17,28,29,30], we assessed whether SMG affects ERK1/2 signaling in cells under SMG. We showed that SMG significantly inhibited phosphorylation of ERK1/2 (T202/Y204) under SMG compared to the control 1 g condition (Figure 6C).

### 2.7. CNF1 Restores SMG-Induced Cellular Morphology and Molecular Signaling Events

**Figure 7:**
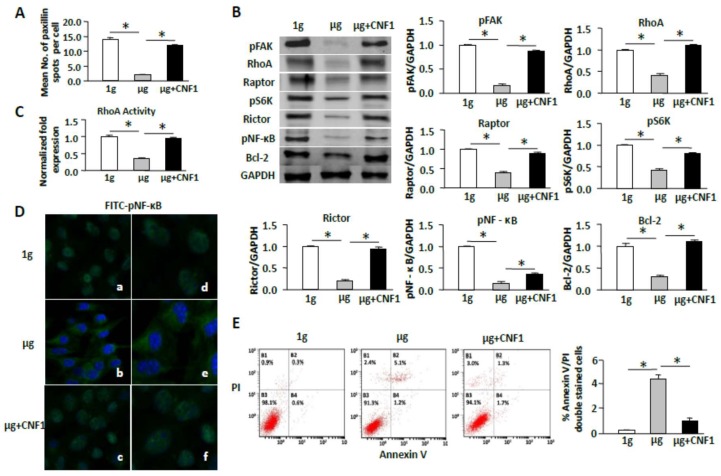
CNF1 restores focal adhesions and NEPCs and activates FAK/RhoA, mTORC1/NF-κB, and ERK1/2 pathways leading to apoptosis reduction in cells under SMG. (**A**) BL6-10 cells were cultured in chamber slides for one day at 1 g, µg, and µg + CNF1, and cells were stained with anti-paxillin, and paxillin spots were counted for each cell. Approximately 20 cells were analyzed per experimental condition; (**B**) Lysates were harvested from BL6-10 cells cultured for two days under 1 g or µg and subjected to SDS-PAGE analysis. Proteins were transferred onto PVDF membranes. Blots were stained with various antibodies and analyzed by chemiluminescence. Bands were qualified using Imaging Lab software (Bio-Rad). Densitometric values were normalized to the GAPDH control; (**C**) RhoA activity analysis. BL6-10 cells were cultured for three days at 1 g, µg, and µg + CNF1 and were subjected to RhoA activity assay by using G-LISA RhoA Activation Assay Biochem kit. Data represent the mean ± SD of three independent experiments; (**D**) BL6-10 cells cultured for three days at 1 g or µg in a Lab-Tek1 II Chamber Slide^TM^ System were fixed with paraformaldehyde, and subsequently incubated with anti-p-NF-κB (S337) (green) antibody and then incubated with FITC-labeled goat-anti-rabbit secondary antibody. Slides were covered using Prolong Gold Antifade Reagent with DAPI (blue) and observed by confocal microscopy. Panels (**a**–**c**) using 20× magnification; panels (**d**–**f**) using 50× magnification; (**E**) BL6-10 tumor cells cultured under ground conditions (1 g) or SMG (µg) and SMG (µg) + CNF1 for one day were stained with Annexin V-FITC and propidium iodide (PI), and then analyzed by flow cytometry. * *p* < 0.05 versus different groups. One representative experiment of three is shown.

Since toxin CNF1 from *Escherichia coli* has been found to increase cell focal adhesions via activation of FAK [22] and to activate Rho family members (RhoA, Rac1, and Cdc42) [31,32], we assessed whether CNF1 is able to rescue the observed SMG-induced effects on cellular morphology and molecular signaling. Interestingly, our experiments did show that CNF1 (i) restored focal adhesions (Figure 7A); (ii) enhanced FAK/RhoA signaling (Figure 7B) and RhoA activity (Figure 7C); (iii) activated the mTORC1/NF-κB pathway by up-regulation of mTORC-comprising Raptor and Rictor and mTORC1-regulated pS6K (S235), and pNF-κB (S337) and pNF-κB-regulated Bcl2 (Figure 7B); and (iv) switched cellular localization of FITC-pNF-κB (S337) (green) from the cytoplasm to the nucleus (DAPI, blue) (Figure 7D) in cells under SMG. In addition, CNF1 also (i) restored the formation of NEPCs (Figure 6A); (ii) up-regulated the expression of NEPs (lamin-A, emerin, sun1, and nesprin-3) (Figure 6B); and (iii) activated the ERK1/2 pathway in cells under SMG (Figure 6C). Finally, we performed experiments to assess whether CNF1 affects SMG-enhanced cell apoptosis, and found that administration of CNF1 significantly reduced SMG-enhanced apoptosis in cells under SMG (Figure 7E). Together, our data shows that CNF1 restores focal adhesions and NEPCs and activates FAK/RhoA, mTORC1/NF-κB, and ERK1/2 pathways, leading to reduced apoptosis in cells under SMG. 

## 3. Discussion

Studies showed that SMG altered tumor cell cytoskeleton structure [13,19,20]. However, its molecular mechanism is not well known. In this study, we investigated the effect of SMG on the alteration of BL6-10 cell cytoskeleton structure. We demonstrate that SMG alters the cell cytoskeleton by losing most stress fibers and lamellipodia, consistent with our previous report [2]. To assess the formation of cell focal adhesions, we stained the cells on chamber slides cultured under 1 g or µg conditions with an antibody against paxillin (a focal adhesion-associated adaptor protein), followed by observation under a fluorescein microscope. Interestingly, we find that SMG significantly reduces the formation of cell focal adhesions leading to the inhibition of the FAK/RhoA pathway, the critical molecular signal controlling the cytoskeleton [10], and inhibits the mTORC2 pathway regulating the cytoskeleton structure via controlling RhoA [33,34,35,36]. Our data, thus, clearly indicates that SMG-induced cytoskeletal alterations result from SMG-inhibited FAK-regulated RhoA as well as SMG-inhibited PI3K-regulated mTORC2 [37]. 

Successful cell apoptosis is coordinated by caspases, a family of cysteine proteases, cleaving cell proteins by targeting aspirate residues [38]. Two major pathways were associated with cell apoptosis, the intrinsic pathway and the extrinsic pathway, which activate the apoptosis initiators caspases 8 and 9 [39], the former induced by cell stresses is regulated by Bcl-2 family members, such as anti- and pro-apoptotic molecules, while the latter was induced by the ligation of extracellular “death” molecules, such as TNF-α or Fas, with their cognate membrane receptors (TNFR and FasL) [40]. SMG was reported to induce cell apoptosis via up- and down-regulated expression of pro-apoptosis and anti-apoptosis molecules [3,4]. We previously showed that SMG promoted cell apoptosis through suppressing the NF-κB pathway, leading to up- and down-regulated pro-apoptosis (caspases 3, 7, 8) and anti-apoptosis (Bcl2 and Bnip3) molecules [2]. However, the up-stream molecules regulating the NF-κB-regulated anti-apoptosis effect in cells under SMG condition is still unclear.

Multiple pathways regulate the formation of cell apoptosis, including mTORC, NF-κB, and ERK1/2 [6]. SMG was reported to inhibit cell focal adhesions [19]. However, the significance of SMG-induced inhibition of focal adhesions was not elucidated. We have recently demonstrated that SMG reduced focal adhesions leading to the inhibition of the FAK/RhoA and mTORC1 pathways [22]. In this study, we confirm our above findings that SMG inhibits the formation of focal adhesions and suppresses the FAK and FAK-regulated RhoA family member (RhoA, Rac1, and Cdc42) signaling, as well as suppresses the RhoA-controlled mTORC1 (S6K and EIF4E) pathway. It has been reported that mTORC1 regulates NF-κB controlling cell apoptosis via up-regulation of the anti-apoptosis Bcl2 molecule [26], while SMG inhibits the NF-κB pathway [41]. We assume that SMG-induced apoptosis may be derived from the SMG-inhibited FAK/RhoA-regulated mTORC1/NF-κB pathway. To address this assumption, we assessed the expression of the mTORC1/NF-κB pathway-related molecules in cells under SMG. We demonstrate that SMG down-regulates expression of not only the mTORC1 pathway-related pS6K (S235) and pEIF4E (S209), but also the NF-κB pathway-related pNF-κB (S337) and Bcl2. In addition, SMG also switches cellular localization of pNF-κB (S337) from the nucleus to the cytoplasm. Furthermore, we demonstrate that rapamycin, the inhibitor of mTORC1, also promotes apoptosis via down-regulation of the mTORC1/NF-κB pathway in cells under 1 g conditions. Taken together, our data indicate that SMG promotes cell apoptosis via down-regulation of the mTORC1/NF-κB pathway.

**Figure 8:**
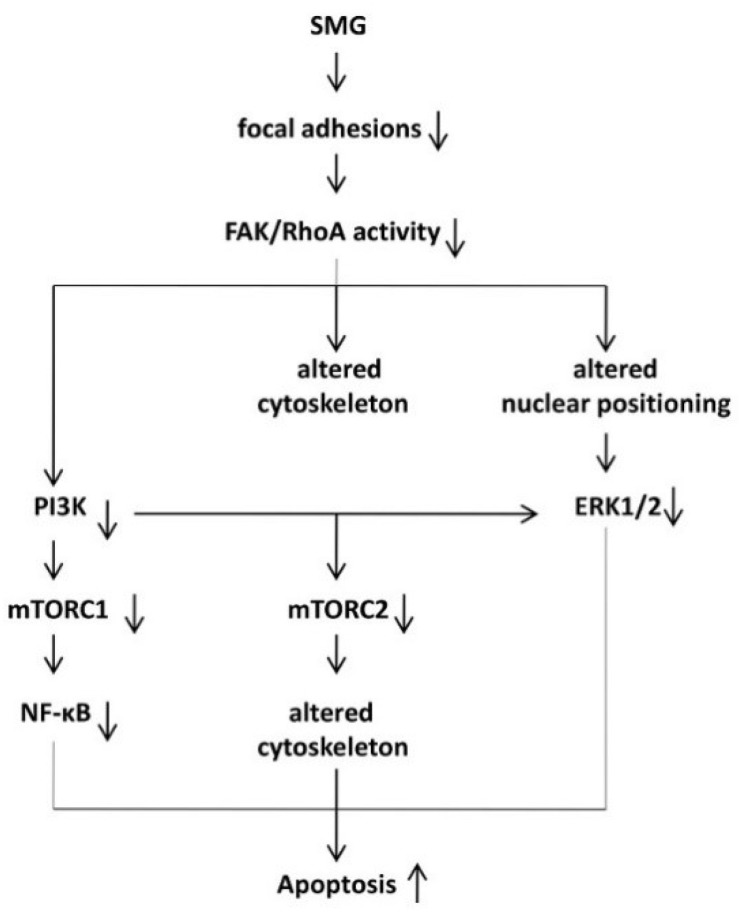
Schematic diagram presenting pathways where simulated microgravity reduces focal adhesions and alters the cytoskeleton and nuclear positioning, leading to enhanced cell apoptosis via suppressing FAK/RhoA-regulated mTORC1/NF-κB and ERK1/2 pathways pathways (solid up-arrows: enhance; solid down-arrows: reduce).

To confirm the above findings, we performed SMG studies using CNF1, a broad-spectrum activator of FAK and Rho proteins [22,31,32]. In this study, we demonstrate that the CNF1 toxin reduces apoptosis in cells under SMG. We also show that CNF1 activates the upstream signaling (FAK and RhoA) of the mTORC1 pathway and is capable of converting the SMG-induced reduction of cell focal adhesions and SMG-induced FAK/RhoA-regulated inhibition of the mTORC1/NF-κB pathway. Therefore, our data confirm that SMG reduces focal adhesions, leading to enhanced cell apoptosis via suppressing the FAK/RhoA-regulated mTORC1/NF-κB pathway (Figure 8).

The nuclear envelope is a double-layer membrane separating nuclear chromatin from the cytoplasm, thereby conferring regulation of gene expression and DNA replication [42]. The outer nuclear membrane (ONM) is contiguous with the cytoplasmic reticulum, while the inner nuclear membrane (INM) contains unique integral NEPs (such as sun1, emerin, lamin A) linking a network of nuclear lamins, called the nucleoskeleton (intermediate filaments), that underlies the INM in the nucleoplasm, and links the cytoskeleton in the cytoplasm through the nuclear envelope connectors (such as nesprins) [16]. NEPs that comprise both nuclear envelope proteins and connectors play an important role in the regulation of nuclear positioning, which affects cytoplasmic signaling pathways [17,18,28,29,43,44]. In this study, we, for the first time, demonstrate that SMG inhibits the expression of NEPs (sun1, emerin, lamin A, and nesprins), and alters nuclear positioning. It has been demonstrated that FAK controls cell survival and mediates cell apoptosis via the ERK1/2 signaling pathway [14], and alteration of nuclear positioning also regulates ERK1/2 [16]. In this study, we assessed whether SMG reduced FAK activity and altered nuclear positioning affected ERK1/2 signaling. Our experiments show that SMG reduces the phosphorylation of ERK1/2 (T202/Y204). Therefore, our data indicate that SMG reduces focal adhesions and promotes cell apoptosis via down-regulation of NEPs and alteration of nuclear positioning leading to down-regulation of the ERK1/2 pathway.

The cytoskeleton alteration has been reported to induce cell apoptosis [45]. Especially, the dynamics of actin and the expression of ABPs (such as down- and up-regulation of F-actin filamin-A and ABP TM1) directly affecting mitochondrial membrane permeabilization and caspase activation make a significant contribution to apoptosis [23]. The mTORC2 signaling has been reported to alter the cytoskeleton structure via suppressing Rho GTPases with a regulatory effect on the expression of ABPs [30]. In this study, we also assessed whether SMG affects the expression of ABP TM1 [46] and F-actin filamin-A [47], and whether SMG affects the mTORC2 pathway. We demonstrate that SMG down- and up-regulates the expression of F-actin filamin-A and ABP TM1, respectively, which is consistent with its promotion of apoptosis [23], and suppresses the mTORC2 pathway in cells under SMG, indicating that SMG promotes cell apoptosis via altered cytoskeleton-induced alteration of actin dynamics and the suppression of the PI3K-regulated mTORC2 pathway. 

To confirm the above findings, we performed SMG studies using the FAK/RhoA activator CNF1 [31,32] in cells under SMG. We demonstrate that administration of CNF1 not only restores SMG-altered nuclear positioning by up-regulation of NEPs and SMG-inhibited ERK1/2 pathway, but also restores SMG-altered actin dynamics by down-regulation of ABP TM1 and up-regulation of the actin filamin-A, and that SMG inhibited the mTORC2 pathway in cells. Therefore, our data confirm that SMG alters the cytoskeleton and nuclear positioning leading to enhanced apoptosis via suppressing the FAK/RhoA-regulated ERK1/2 and mTORC2 pathways (Figure 8).

The aerospace microgravity has been demonstrated to inhibit tumor cell proliferation and metastasis [48], promote cell apoptosis [3,4], and suppress osteoblastic differentiation and mineralization leading to bone loss [49]. However, the underlying mechanisms for these observations are not clearly understood. We, for the first time, have demonstrated that SMG significantly inhibits cell focal adhesions and FAK/RhoA activity, and elucidated that SMG reduces tumor cell proliferation and metastasis via suppressing the FAK/RhoA-controlled mTORC1 pathway [22]. In this study, we further demonstrate that SMG also promotes cell apoptosis through abrogation of cell focal adhesions leading to the suppression of FAK/RhoA-controlled mTORC1/NF-κB and ERK1/2 pathways. We, therefore, assume that the FAK/RhoA regulatory network may be important in other SMG-induced physiological alterations, such as SMG-induced bone loss [49], often seen in rheumatoid arthritis [50]. To assess this assumption, we are currently conducting similar SMG experiments using the MC3T3 pre-osteoblast cell line [49] in our laboratory.

Taken together, our data reveal a new molecular mechanism for SMG-related studies, that SMG reduces focal adhesion complexes and alters the cytoskeleton and nuclear positioning leading to enhanced cell apoptosis via suppressing FAK/RhoA-regulated mTORC1/NF-κB and ERK1/2 pathways. Thus, the FAK/RhoA regulatory network may become an important target for development of new therapeutics for humans under the spaceflight environment with stressed physiological challenges and for other human diseases. 

## 4. Materials and Methods

### 4.1. Cells, Antibodies, and Reagents

BL6-10, which is a highly lung-metastatic B16 melanoma cell line [22], and showed enhanced cell apoptosis under SMG [2], was maintained in α-MEM medium supplemented with 10% fetal calf serum (FCS). Rabbit antibodies against Raptor and Rictor were purchased from Thermo Fisher Scientific (Rockford, IL, USA). Rabbit antibodies against RhoA and Rac1 were purchased from Santa Cruz Biotechnology (Dallas, TX, USA). Rabbit antibodies against Cdc42, FAK, phosphor-FAK (pFAK; Y397), pS6K (S235), pERK1/2 (T202/Y204), pNF-κB (S337), and Bcl2 were obtained from Cell Signaling Technology (Boston, MA, USA). Rabbit antibodies against paxillin, vinculin, lamin-A, emerin, sun1, filament-A, nesprin-3, and tropomyosin-1 (TM1) were obtained from Abcam Inc. (Cambridge, MA, USA). FITC-labeled phalloidin was purchased from Sigma-Aldrich (St. Louis, MO, USA). FITC-Annexin V Apoptosis Detection kit II was purchased from BD Pharmingen™ (Toronto, ON, Canada). Toxin cytotoxic necrotizing factor-1 (CNF1), which activates both FAK and RhoA [22,31,32], was obtained from Harald Genth, Hannover Medical School, Hannover, Germany [31]. FITC-conjugated goat anti-rabbit (ZF-0314) secondary antibody specific for primary antibodies were purchased from ZSGB-Bio Inc. (Beijing, China). The Prolong1gold Antifade Reagent with DAPI was obtained from Life technologies Inc. (Carlsbad, CA, USA). The mTORC inhibitor rapamycin was purchased from SelleckchemInc (Houston, TX, USA).

### 4.2. Clinostat of Simulated Microgravity

The SMG environment [2,22] is modeled by the random positional machine (RPM), which is a three-dimensional clinostat manufactured by the Center for Space Science and Applied Research, Chinese Academy of Sciences (Beijing, China). The RPM consists of two independent rotating frames, an inner frame and an outer frame. Both frames can rotate randomly in three-dimensions with changes in the acceleration and direction of the samples over time, resulting in randomization of the gravitational vector, low fluid shear stress, and three-dimensional spatial freedom. The angular velocity of the rotation is at a speed of 30°/s. To examine the gravitational effect, BL6-10 tumor cells were transferred into T25 culture flasks or chamber culture slides (Nalge Nunc International Inc., Rochester, NY, USA), and grown for 24 h, allowing cell to attach to the culture flasks or chamber slides. Cells were then cultured for one to two days at 37 °C in a CO_2_ incubator under ground conditions (1 g) or on the clinostat under SMG conditions (µg) with the chambers or flasks filled up with 37 °C warm culture medium [2]. To assess the effect of CNF1, we added CNF1 (30 ng/mL) to BL6-10 cells [31,32] under SMG.

### 4.3. Fluorescent Microscopy

For microtubule immunofluorescence staining, after removing the medium, BL6-10 cells were washed twice with PBS, fixed in 4% paraformaldehyde at room temperature for 15 min. After washing twice with PBS, the cells were permeabilized in PBS containing 0.5% Triton X-100 for 10 min and blocking in 1% BSA in PBS at room temperature for 30 min. The cells were incubated with 1:25 monoclonal anti-β-tubulin-FITC diluted in PBS containing 1% BSA for 1 h in dark at room temperature. For microfilament fluorescence staining, the permeabilized cells were incubated with 1:20 FITC-labeled phalloidin diluted in PBS for 30 min in the dark at room temperature. For measurement of cell focal adhesions, chamber slides were used to grow BL6-10 cells (Nalge Nunc International Inc.), and the permeabilized cells were incubated with anti-paxillin or anti-vinculin antibody (1:100 diluted in PBS) containing 1% BSA for 24 h at 4 °C overnight, followed by using FITC- and PE-labeled anti-rabbit antibody, respectively. After rinsing three times with PBS, plastic chambers were removed, and slides covered with cover slips for fluorescence microscopy [2].

### 4.4. Western Blotting Analysis

Cells were harvested and washed twice in ice-cold PBS, then lysed in lysis buffer containing 1% NP40, 0.5% sodium deoxycholate, and 0.1% SDS in PBS, supplemented with protease and phosphatase inhibitors, for 30 min on ice with gentle stirring. The lysates were centrifuged and the supernatant was collected. For Western bloting, 30–50 µg total protein sample was loaded into each well of the 10% SDS-PAGE gel. After electrophoresis, samples were electrotransferred onto a 0.22 μm polyninylidene fluoride (PVDF) membrane (Millipore, Middlesex County, MA, USA). After blocking with 5% skimmed milk powder in TBST (pH 7.4, TBS with 0.1% Tween-20), membranes were incubated with various primary antibodies overnight in 4 °C. After washing, blots were incubated with suitable secondary antibodies conjugated to horseradish peroxidase. The protein bands developed with horseradish peroxidase developer solution were quantified using chemiluminescence [2]. Glyceraldehyde 3-phosphate dehydrogenase (GAPDH) was used as the internal reference.

### 4.5. Confocal Microscopy

BL6-10 cells were seeded into wells of a Lab-Tek1 II Chamber Slide System (Nalge Nunc International Inc.). After BL6-10 cells were cultured in α-MEM with 10% FBS under 1 g for 24 h. The chambers were filled with the above culture medium to avoid the presence of air bubbles, sealed, placed at the center of the inner frame, and then clinorotated at 37 °C for 24 h in a CO_2_ incubator. The control cells (1 g) were just placed in the incubator. For pNF-κB (S337) staining, BL6-10 cells were washed twice with PBS, the cells were permeabilized in PBS containing 0.5% Triton X-100 for 10 min and blocked in 1% BSA in PBS at room temperature for 30 min. The cells were incubated with rabbit anti-pNF-κB (S337) antibody containing 1% BSA for 2 h, and then incubated with FITC-labeled goat-anti-rabbit secondary antibody for 1 h in the dark at room temperature. After rinsing three times with PBS, the slides were covered with Prolong Gold Antifade Reagent with DAPI and observed by confocal microscopy [2].

### 4.6. Annexin-V and Propidium Iodide Staining

BL6-10 cells were stained with Annexin-V and propidium iodide (PI) using FITC-Annexin V Apoptosis Detection kit II (BD Pharmingen™, ON, Canada) according to the protocol of the company [2]. 

### 4.7. RhoA Activity Assaay

To measure RhoA activity and cell adhesion, we performed in vitro experiments using a G-LISA RhoA Activation Assay Biochem kit (Cytoskeleton Inc., Denver, CO, USA) and a CytoSelect™ 24-Well Cell Adhesion Assay kit, according to the manufacturers’ manuals [22].

### 4.8. Statistical Analysis

Statistical analysis was conducted using Graphpad Prism-3.0 (GraphPad Software, San Diego, CA, USA), and statistical significance among groups was analyzed using the student *t* test [2]. *p* values < 0.05 were considered statistically significant.

## Abbreviations

Cdc42	Cell division-control protein-42
CNF1	Cytotoxic necrotizing factor-1
EIF4E	Eukaryotic initiation factor 4E
ERK1/2	Extracellular signal-regulated kinase-1/2
FAK	Focal adhesion kinase
FITC	Fluorescein isothiocyanate
GAPDH	Glyceraldehyde 3-phosphate dehydrogenase
mTORC1	Mammalian target of rapamycin complex-1
NF-Κb	Nuclear factor-kappa B
NEPC	Nuclear envelope protein complex
PI	Propidium iodide
Rac1	Ras-related C3 botulinum-toxin substrate-1
S6K	S6 kinase
RhoA	Ras homolog gene-family member-A
RPM	Random positional machine
SMG	Simulated microgravity

## References

[1] Williams D., Kuipers A., Mukai C., Thirsk R. (2009). Acclimation during space flight: Effects on human physiology. Can. Med. Assoc. J..

[2] Zhao T., Tang X., Umeshappa C.S., Ma H., Gao H., Deng Y., Freywald A., Xiang J. (2016). Simulated Microgravity Promotes Cell Apoptosis Through Suppressing Uev1A/TICAM/TRAF/NF-κB-Regulated Anti-Apoptosis and p53/PCNA- and ATM/ATR-Chk1/2-Controlled DNA-Damage Response Pathways. J. Cell. Biochem..

[3] Kossmehl P., Shakibaei M., Cogoli A., Infanger M., Curcio F., Schönberger J., Eilles C., Bauer J., Pickenhahn H., Schulze-Tanzil G. (2003). Weightlessness induced apoptosis in normal thyroid cells and papillary thyroid carcinoma cells via extrinsic and intrinsic pathways. Endocrinology.

[4] Kumari R., Singh K.P., DuMond J.W. (2009). Simulated microgravity decreases DNA repair capacity and induces DNA damage in human lymphocytes. J. Cell. Biochem..

[5] Wyllie A.H. (1997). Apoptosis: An overview. Br. Med. Bull..

[6] Wang J., Liew O.W., Richards A.M., Chen Y.-T. (2016). Overview of microRNAs in cardiac hypertrophy, fibrosis, and apoptosis. Int. J. Mol. Sci..

[7] Wickstead B., Gull K. (2011). The evolution of the cytoskeleton. J. Cell Biol..

[8] Geiger B., Spatz J.P., Bershadsky A.D. (2009). Environmental sensing through focal adhesions. Nat. Rev. Mol. Cell Biol..

[9] Sulzmaier F.J., Jean C., Schlaepfer D.D. (2014). FAK in cancer: Mechanistic findings and clinical applications. Nat. Rev. Cancer.

[10] Hall A. (1998). Rho GTPases and the actin cytoskeleton. Science.

[11] Gordon B.S., Kazi A.A., Coleman C.S., Dennis M.D., Chau V., Jefferson L.S., Kimball S.R. (2014). RhoA modulates signaling through the mechanistic target of rapamycin complex 1 (mTORC1) in mammalian cells. Cell. Signal.

[12] Ghasemi A., Hashemy S.I., Aghaei M., Panjehpour M. (2017). RhoA/ROCK pathway mediates leptin-induced uPA expression to promote cell invasion in ovarian cancer cells. Cell. Signal..

[13] Vassy J., Portet S., Beil M., Millot G., Fauvel-Lafeve F., Karniguian A., Gasset G., Irinopoulou T., Calvo F., Rigaut J. (2001). The effect of weightlessness on cytoskeleton architecture and proliferation of human breast cancer cell line MCF-7. FASEB J..

[14] Huang D., Khoe M., Befekadu M., Chung S., Takata Y., Ilic D., Bryer-Ash M. (2007). Focal adhesion kinase mediates cell survival via NF-κB and ERK signaling pathways. Am. J. Physiol. Cell Physiol..

[15] Konstantinidou G., Ramadori G., Torti F., Kangasniemi K., Ramirez R.E., Cai Y., Behrens C., Dellinger M.T., Brekken R.A., Wistuba I.I. (2013). RHOA-FAK Is a Required Signaling Axis for the Maintenance of KRAS-Driven Lung Adenocarcinomas. Can. Discov..

[16] Gundersen G.G., Worman H.J. (2013). Nuclear positioning. Cell.

[17] Muchir A., Pavlidis P., Decostre V., Herron A.J., Arimura T., Bonne G., Worman H.J. (2007). Activation of MAPK pathways links LMNA mutations to cardiomyopathy in Emery-Dreifuss muscular dystrophy. J. Clin. Investig..

[18] Hale C.M., Shrestha A.L., Khatau S.B., Stewart-Hutchinson P., Hernandez L., Stewart C.L., Hodzic D., Wirtz D. (2008). Dysfunctional connections between the nucleus and the actin and microtubule networks in laminopathic models. Biophys. J..

[19] Hughes-Fulford M. (2003). Function of the cytoskeleton in gravisensing during spaceflight. Adv. Space Res..

[20] Infanger M., Kossmehl P., Shakibaei M., Bauer J., Kossmehl-Zorn S., Cogoli A., Curcio F., Oksche A., Wehland M., Kreutz R. (2006). Simulated weightlessness changes the cytoskeleton and extracellular matrix proteins in papillary thyroid carcinoma cells. Cell Tissue Res..

[21] Lewis M.L., Reynolds J.L., Cubano L.A., Hatton J.P., Lawless B.D., Piepmeier E.H. (1998). Spaceflight alters microtubules and increases apoptosis in human lymphocytes (Jurkat). FASEB J..

[22] Tan X., Xu A., Zhao T., Zhao Q., Zhang J., Fan C., Deng Y., Freywald A., Genth H., Xiang J. (2018). Simulated microgravity inhibits cell focal adhesions leading to reduced melanoma cell proliferation and metastasis via FAK/RhoA-regulated mTORC1 and AMPK pathways. Sci. Rep..

[23] Desouza M., Gunning P.W., Stehn J.R. (2012). The actin cytoskeleton as a sensor and mediator of apoptosis. Bioarchitecture.

[24] Del Re D.P., Miyamoto S., Brown J.H. (2008). Focal Adhesion Kinase as a RhoA-activable Signaling Scaffold Mediating Akt Activation and Cardiomyocyte Protection. J. Biol. Chem..

[25] Chi H. (2012). Regulation and function of mTOR signalling in T cell fate decisions. Nat. Rev. Immunol..

[26] Barkett M., Gilmore T.D. (1999). Control of apoptosis by Rel/NF-κB transcription factors. Oncogene.

[27] Giordano A., Avellino R., Ferraro P., Romano S., Corcione N., Romano M.F. (2006). Rapamycin antagonizes NF-κB nuclear translocation activated by TNF-α in primary vascular smooth muscle cells and enhances apoptosis. Am. J. Physiol. Heart Circ. Physiol..

[28] Muchir A., Wu W., Worman H.J. (2009). Reduced expression of A-type lamins and emerin activates extracellular signal-regulated kinase in cultured cells. Biochim. Biophys. Acta Mol. Basis Dis..

[29] González J.M., Navarro-Puche A., Casar B., Crespo P., Andrés V. (2008). Fast regulation of AP-1 activity through interaction of lamin A/C, ERK1/2, and c-Fos at the nuclear envelope. J. Cell Biol..

[30] Liu L., Luo Y., Chen L., Shen T., Xu B., Chen W., Zhou H., Han X., Huang S. (2010). Rapamycin Inhibits Cytoskeleton Reorganization and Cell Motility by Suppressing RhoA Expression and Activity. J. Biol. Chem..

[31] May M., Kolbe T., Wang T., Schmidt G., Genth H. (2012). Increased cell-matrix adhesion upon constitutive activation of Rho proteins by cytotoxic necrotizing factors from *E. coli* and *Y. pseudotuberculosis*. J. Signal Transduct..

[32] Fabbri A., Travaglione S., Fiorentini C. (2010). Escherichia coli cytotoxic necrotizing factor 1 (CNF1): Toxin biology, in vivo applications and therapeutic potential. Toxins.

[33] Sarbassov D.D., Guertin D.A., Ali S.M., Sabatini D.M. (2005). Phosphorylation and Regulation of Akt/PKB by the Rictor-mTOR Complex. Science.

[34] Goncharova E.A., Goncharov D.A., Li H., Pimtong W., Lu S., Khavin I., Krymskaya V.P. (2011). mTORC2 Is Required for Proliferation and Survival of TSC2-Null Cells. Mol. Cell. Biol..

[35] Facchinetti V., Ouyang W., Wei H., Soto N., Lazorchak A., Gould C., Lowry C., Newton A.C., Mao Y., Miao R.Q. (2008). The mammalian target of rapamycin complex 2 controls folding and stability of Akt and protein kinase C. EMBO J..

[36] Ikenoue T., Inoki K., Yang Q., Zhou X., Guan K.L. (2008). Essential function of TORC2 in PKC and Akt turn motif phosphorylation, maturation and signalling. EMBO J..

[37] Thomson A.W., Turnquist H.R., Raimondi G. (2009). Immunoregulatory functions of mTOR inhibition. Nat. Rev. Immunol..

[38] Fuentes-Prior P., Salvesen G.S. (2004). The protein structures that shape caspase activity, specificity, activation and inhibition. Biochem. J..

[39] Ye Z., Shi M., Xu S., Xiang J. (2010). LFA-1 defect-induced effector/memory CD8+ T cell apoptosis is mediated via Bcl-2/Caspase pathways and associated with downregulation of CD27 and IL-15R. Mol. Immunol..

[40] Taylor R.C., Cullen S.P., Martin S.J. (2008). Apoptosis: Controlled demolition at the cellular level. Nat. Rev. Mol. Cell Biol..

[41] Chang T.T., Walther I., Li C.F., Boonyaratanakornkit J., Galleri G., Meloni M.A., Pippia P., Cogoli A., Hughes-Fulford M. (2012). The Rel/NF-κB pathway and transcription of immediate early genes in T cell activation are inhibited by microgravity. J. Leukoc. Biol..

[42] Kiseleva E., Goldberg M.W., Cronshaw J., Allen T.D. (2000). The nuclear pore complex: Structure, function, and dynamics. Crit. Rev. Eukaryot. Gene Expr..

[43] Folker E.S., Östlund C., Luxton G.W.G., Worman H.J., Gundersen G.G. (2011). Lamin A variants that cause striated muscle disease are defective in anchoring transmembrane actin-associated nuclear lines for nuclear movement. Proc. Natl. Acad. Sci. USA.

[44] Warren D.T., Tajsic T., Mellad J.A., Searles R., Zhang Q., Shanahan C.M. (2010). Novel Nuclear Nesprin-2 Variants Tether Active Extracellular Signal-regulated MAPK1 and MAPK2 at Promyelocytic Leukemia Protein Nuclear Bodies and Act to Regulate Smooth Muscle Cell Proliferation. J. Biol. Chem..

[45] Ndozangue-Touriguine O., Hamelin J., Bréard J. (2008). Cytoskeleton and apoptosis. Biochem. Pharmacol..

[46] Bharadwaj S., Thanawala R., Bon G., Falcioni R., Prasad G.L. (2005). Resensitization of breast cancer cells to anoikis by Tropomyosin-1: Role of Rho kinase-dependent cytoskeleton and adhesion. Oncogene.

[47] Umeda T., Kouchi Z., Kawahara H., Tomioka S., Sasagawa N., Maeda T., Sorimach H., Ishiura S., Suzuki K. (2001). Limited Proteolysis of Filamin Is Catalyzed by Caspase-3 in U937 and Jurkat Cells. J. Biochem..

[48] Chang D., Xu H., Guo Y., Jiang X., Liu Y., Li K., Pan C., Yuan M., Wang J., Li T. (2013). Simulated microgravity alters the metastatic potential of a human lung adenocarcinoma cell line. Cell. Dev. Biol. Anim..

[49] Seicho M., Yumi K., Louis Y., Yuichi M., Hiroki N. (2008). Impact of the microgravity environment in a 3-dimensional clinostat on osteoblast- and osteoclast-like cells. Cell Biol. Int..

[50] Hardy R., Cooper M.S. (2009). Bone loss in inflammatory disorders. J. Endocrinol..

